# From Gestational Diabetes to Type 2 Diabetes—Is Poor Sleep to Blame?

**DOI:** 10.1001/jamanetworkopen.2025.0149

**Published:** 2025-03-03

**Authors:** Christian Benedict, Marie-Pierre St-Onge

**Affiliations:** Department of Pharmaceutical Biosciences, Uppsala University, Uppsala, Sweden; Center of Excellence for Sleep and Circadian Research, Department of Medicine, Columbia University Irving Medical Center, New York, New York; Division of General Medicine, Department of Medicine, Columbia University Irving Medical Center, New York, New York

Gestational diabetes is a condition characterized by glucose intolerance first detected during pregnancy in which blood glucose levels are elevated but do not meet the criteria for overt diabetes. Gestational diabetes affects approximately 14% of pregnancies worldwide, with its prevalence varying based on factors such as race and ethnicity, age, obesity, and diagnostic criteria.^1^ One of the most concerning long-term consequences of gestational diabetes for women is the significantly elevated risk of developing type 2 diabetes in the years following pregnancy. For example, a study of 50 884 women initially without type 2 diabetes revealed that having a history of 1 or more pregnancies complicated by gestational diabetes was associated with an increased age-specific risk of developing type 2 diabetes by 287% in the 6 to 15 years after the affected pregnancy.^2^ Although the hazard ratio decreased by approximately 24% per decade, the risk remained elevated for more than 35 years following pregnancy. Given this long-term risk, regular screening for type 2 diabetes is highly advisable for women with a history of gestational diabetes even decades after pregnancy.

Poor sleep health has emerged as a significant factor associated with an increased risk of type 2 diabetes in adulthood. A study involving 247 867 middle-aged participants, 52.3% of whom were female, found that individuals who slept 5 hours or less per night had a 16% to 41% higher risk of developing type 2 diabetes compared with those who habitually slept between 7 and 8 hours per night.^3^ Experimental studies further strengthened the connection between short sleep duration and glucose regulation. For example, in a study in which women aged 20 to 75 years were restricted to less than 7 hours of sleep per night for 6 weeks, researchers observed impaired insulin sensitivity, with these effects being more pronounced in postmenopausal women compared with premenopausal women.^4^ In addition to sleep duration, sleep disturbances, such as frequent snoring, a key symptom of obstructive sleep apnea, have also been associated with an increased risk of type 2 diabetes. This was demonstrated in a study of 69 852 female nurses aged 40 to 65 years that found that compared with nonsnorers, women who snored occasionally had a 41% increased relative risk, while those who snored regularly had a 103% increased relative risk of developing type 2 diabetes over 10 years.^5^ Taken together, these findings highlight the pivotal role of sleep health both in terms of duration and quality in influencing the risk of developing type 2 diabetes in women.

Given these associations of gestational diabetes and sleep quality with risk of type 2 diabetes, Yin and colleagues^6^ explored whether the risk of developing type 2 diabetes in women who experienced gestational diabetes was associated with sleep quality (duration, snoring, and daytime sleepiness). Women with a history of gestational diabetes who participated in the Nurses’ Health Study II were followed up over a mean duration of 17.3 years. The study showed that women with a history of gestational diabetes who snored occasionally to regularly or slept fewer than 7 hours per day had significantly higher risks of developing type 2 diabetes (54% to 61% higher risk, respectively) compared with those who almost never snored or slept 6 or fewer hours per day (32% higher risk compared with women who slept 7 to 8 hours per day). Women who expressed daytime sleepiness 4 or more days per week did not have an increased risk of type 2 diabetes after adjustment for multiple covariates. Additionally, it is important to evaluate whether these sleep disturbances, namely short sleep duration and snoring, have additive influences on risk of type 2 diabetes. Yin and colleagues^6^ found that among women with a gestational diabetes history, those who both slept 6 hours or less per day and snored either occasionally or regularly had approximately twice the risk of developing type 2 diabetes compared with those who slept 7 to 8 hours per day and rarely or never snored. These findings underscore the critical need to incorporate sleep health into a comprehensive strategy aimed at reducing long-term type 2 diabetes risk, especially for women with a history of gestational diabetes.

Other research involving women suggests that short sleep duration can enhance the brain’s response to calorie-dense food stimuli,^7^ potentially promoting weight gain over time—a well-established risk factor for the development of type 2 diabetes.^8^ Similarly, snoring, often associated with increased respiratory efforts during sleep, has been shown to activate the sympathetic nervous system.^9^ When snoring occurs regularly, this activation may further elevate the risk of type 2 diabetes by impairing glucose metabolism.

While mechanisms associated with short sleep duration and regular snoring are likely to contribute to an increased risk of type 2 diabetes, it is crucial to determine whether women with a history of gestational diabetes have a particularly high risk of impaired glucose metabolism compared with women without such a history. For example, is the enhanced brain response to calorie-dense foods caused by insufficient sleep^7^ or the activation of the sympathetic nervous system associated with snoring^9^ more pronounced in women with a gestational diabetes history? Identifying this susceptibility could provide crucial insights into how gestational diabetes history may interact with sleep health to elevate the risk of type 2 diabetes.

To conclude, while gestational diabetes is already a known risk factor for the development of type 2 diabetes later in life,^2^ the findings of Yin and colleagues^6^ suggest that poor sleep health may further exacerbate this risk. Regular screening of long-term glycemic control should be accompanied by questions regarding sleep duration and quality to identify those at greatest risk of developing type 2 diabetes in the long term. Proactive management of sleep disturbances in this at-risk population could provide an important opportunity to mitigate the risk of type 2 diabetes onset. Given the observational nature of the study by Yin and colleagues^6^ and the lack of objective and frequent measures of sleep during the follow-up period, future studies should be conducted to test causality. Investigations into the underlying biological mechanisms are crucial to better understand how poor sleep health contributes to the development of type 2 diabetes in women with a history of gestational diabetes.

## References

[1] Sweeting A, Hannah W, Backman H, Epidemiology and management of gestational diabetes. Lancet. 2024;404(10448):175–192. doi:10.1016/S0140-6736(24)00825-0 38909620

[2] Diaz-Santana MV, O’Brien KM, Park YM, Sandler DP, Weinberg CR. Persistence of risk for type 2 diabetes after gestational diabetes mellitus. Diabetes Care. 2022;45(4):864–870. doi:10.2337/dc21-1430 35104325 PMC9016728

[3] Nôga DA, Meth EMES, Pacheco AP, Habitual short sleep duration, diet, and development of type 2 diabetes in adults. JAMA Netw Open. 2024;7(3):e241147. doi:10.1001/jamanetworkopen.2024.1147 38441893 PMC10915681

[4] Zuraikat FM, Laferrère B, Cheng B, Chronic insufficient sleep in women impairs insulin sensitivity independent of adiposity changes: results of a randomized trial. Diabetes Care. 2024;47(1):117–125. doi:10.2337/dc23-1156 37955852 PMC10733650

[5] Al-Delaimy WK, Manson JE, Willett WC, Stampfer MJ, Hu FB. Snoring as a risk factor for type II diabetes mellitus: a prospective study. Am J Epidemiol. 2002;155(5):387–393. doi:10.1093/aje/155.5.387 11867347

[6] Yin X, Bao W, Ley SH, Sleep characteristics and long-term risk of type 2 diabetes among women with gestational diabetes. JAMA Netw Open. 2025;8(3):e250142. doi:10.1001/jamanetworkopen.2025.0142 40042841 PMC11883505

[7] St-Onge MP, McReynolds A, Trivedi ZB, Roberts AL, Sy M, Hirsch J. Sleep restriction leads to increased activation of brain regions sensitive to food stimuli. Am J Clin Nutr. 2012;95(4):818–824. doi:10.3945/ajcn.111.027383 22357722 PMC3302360

[8] Colditz GA, Willett WC, Rotnitzky A, Manson JE. Weight gain as a risk factor for clinical diabetes mellitus in women. Ann Intern Med. 1995;122(7):481–486. doi:10.7326/0003-4819-122-7-199504010-00001 7872581

[9] Jennum P, Schultz-Larsen K, Christensen N. Snoring, sympathetic activity and cardiovascular risk factors in a 70 year old population. Eur J Epidemiol. 1993;9(5):477–482. doi:10.1007/BF00209524 8307131

